# Serum BDNF levels correlate with regional cortical thickness in minor depression: a pilot study

**DOI:** 10.1038/s41598-020-71317-y

**Published:** 2020-09-03

**Authors:** M. Polyakova, F. Beyer, K. Mueller, C. Sander, V. Witte, L. Lampe, F. Rodrigues, S. Riedel-Heller, J. Kratzsch, K. T. Hoffmann, A. Villringer, P. Schoenknecht, M. L. Schroeter

**Affiliations:** 1 Max Planck Institute for Human Cognitive and Brain Sciences, Leipzig, Germany; 2 Clinic for Psychiatry and Psychotherapy, University of Leipzig, Leipzig, Germany; 3 LIFE—Leipzig Research Center for Civilization Diseases, University of Leipzig, Leipzig, Germany; 4 Institute of Social Medicine, Occupational Health and Public Health (ISAP), Leipzig University, Leipzig, Germany; 5 Institute of Laboratory Medicine, Clinical Chemistry and Molecular Diagnostics, Leipzig University, Leipzig, Germany; 6 Department of Neuroradiology, Leipzig University, Leipzig, Germany; 7 Berlin School of Mind and Brain and the Mind-Brain Institute, Humboldt-University of Berlin, Berlin, Germany; 8 Department of Psychiatry and Psychotherapy, University Affiliated Hospital Arnsdorf, Technical University of Dresden, Dresden, Germany; 9 Clinic for Cognitive Neurology, University of Leipzig, Leipzig, Germany; 10 CRC Obesity Mechanisms, Subproject A1, University of Leipzig, Leipzig, Germany

**Keywords:** Depression, Biomarkers

## Abstract

Serum brain-derived neurotrophic factor (BDNF) reflects state changes in mood disorders. But its relation to brain changes in depression has rarely been investigated in humans. We assessed the association between serum BDNF, cortical thickness, or gray matter volume in 20 subjects with a minor depressive episode and 40 matched healthy subjects. Serum BDNF positively correlated with cortical thickness and volume in multiple brain regions in the minor depression group: the bilateral medial orbitofrontal cortex and rostral anterior cingulate cortex, left insula, and cingulum, right superior frontal gyrus, and other regions—regions typically affected by major depression. Interestingly, these correlations were driven by subjects with first episode depression. There was no significant association between these imaging parameters and serum BDNF in the healthy control group. Interaction analyses supported this finding. Our findings point to a specific association between serum BDNF and magnetic resonance imaging parameters in first-episode minor depression in a region- and condition-dependent manner. A positive correlation between serum BDNF and structural gray matter estimates was most consistently observed for cortical thickness. We discuss why cortical thickness should be preferred to volumetric estimates for such analyses in future studies. Results of our pilot study have to be proven in future larger-scale studies yielding higher statistical power.

## Introduction

Minor depression is a subclinical depressive state characterized by depressed mood or lack of interest, combined with one to three other depressive symptoms disturbing a patient over two weeks. In later life minor depression becomes more prevalent than major depressive disorder (MDD)^1^. Patients suffering from this have an increased risk of developing MDD^2^ or attempting suicide^3^. The pathophysiology of minor depression remains largely unexplored^4^. Its clinical proximity to MDD makes minor depression a good clinical model for examining the earliest pathophysiological changes in depression. Here one has to differentiate between minor depressive episode and minor depressive disorder. For the diagnosis of minor depressive disorder, in contrast to episode, an exclusion of depression history is crucial^5^.

The neurotrophic hypothesis of depression is highly discussed today. It postulates that mood disorders are related to decreased synthesis of brain-derived neurotrophic factor (BDNF) in the brain resulting in impaired synaptogenesis and neuronal activity^6^. Treatment with antidepressants, on the other hand, increases BDNF secretion in the brain^7^ and in serum^8^, whereas the latter is associated with recovery from depression^9^.

In this study, we investigated whether serum (s)BDNF levels are related to changes in human gray matter parameters in subjects with minor depression and in healthy controls. To our knowledge, very few studies have attempted to relate sBDNF to brain imaging parameters. Some region-of-interest-based analyses revealed a positive correlation between sBDNF and the volume of the hippocampus in healthy subjects^10,11^. Others found no correlation of hippocampal and amygdala volumes with sBDNF, neither in healthy subject^12^ nor in subjects with mood disorders^13^ or schizophrenia^12^. One study did not find any relation of cortical thickness across the brain to sBDNF in healthy subjects and subjects with recurrent MDD^14^, and another reported a negative correlation in patients with schizophrenia^12^.

Histologically, parameters such as gray matter volume and cortical thickness measured by magnetic resonance imaging (MRI) in vivo represent distinct brain features^15^. Gray matter volume is, mathematically, a product of thickness and area, where area has more weight^16,17^. In ontogenesis, cortical surface area is defined by the number of neuronal columns and cortical thickness is defined by the number of neurons within the columns. Moreover, these brain features are related to distinct sets of genes^17^. In neuroimaging studies, the histological underpinnings of imaging parameters are rarely taken into account.

In this perspective, studies on the correlation between sBDNF and MRI parameters lack a systematic approach, investigating different diseases using different analysis methods, with potentially improper parameters. Since cortical thickness and volume are distinct measures of the brain^16^, we performed a systematic whole-brain structural MRI study correlating sBDNF levels to these imaging parameters estimated with FreeSurfer. Due to the neurotrophic effects of BDNF we generally hypothesized a positive correlation between sBDNF and cortical estimates, modified due to the reduction of sBDNF and regional gray matter volume/cortical thickness in depressive disorders. Differences between subjects with or without a history of depression were assessed in an explorative analysis.

## Methods

### Subjects

Twenty subjects satisfying DSM-IV criteria^5^ for minor depressive episode were selected from the database of the population-based LIFE-Adult study^18^. In accordance with Structured Psychiatric Interview for DSM-IV Disorders (SKID), every subject had one to four depressive symptoms for at least two weeks, with depressed mood or loss of interest being one of them. Forty healthy volunteers from the same study were free from depressive symptoms or cognitive impairment and were matched at a 1:2 ratio by sex and age to the subjects with minor depression. The study was carried out in accordance with the Declaration of Helsinki and approved by the Ethics Committee of the University of Leipzig. All participants gave written informed consent.

Mild and major neurocognitive disorders^2–4^ (formerly known as mild cognitive impairment and dementia) were excluded according to DSM-5 diagnostic criteria for mild Neurocognitive Disorder (NCD). These criteria require: (A) presence of subjective cognitive disturbance; (B) objective cognitive decline 1–2 standard deviations (SD) below sex- and age-adjusted norms in at least one of five cognitive domains; (C) preserved activities of daily living according to the Activities of Daily Living scale (ADL); (D) absence of delirium and major psychiatric illness (E).

Cognitive testing was performed using the German version of the Consortium to Establish a Registry for Alzheimer’s Disease (CERAD)-plus test battery and a Stroop test. Specific tests or subtests were assigned to each DSM-5 cognitive domain. With Trail Making Test (TMT)-A and Stroop neutral we evaluated attention, with TMT-B/A and Stroop incongruent/neutral executive function. The word list subtest from the CERAD-plus test battery was used for assessment of learning and memory, figure drawing test was used for the visuo-construction/perception domains. Participants’ scores were compared to normative values adjusted for sex, age, and education, obtained from the Basel memory clinic (www.memoryclinic.ch). A mean deviation from the norms was calculated for each cognitive domain if this domain was assessed with more than one test.”

### BDNF measurement

Blood samples were withdrawn from subjects by venipuncture, between 7:25 and 10:45 in the morning, after an overnight fasting. Serum was prepared using the standard operating procedures. In brief, samples were left for 45 min for clotting, followed by a centrifugation step (10 min, 2,750 *g*, 15 °C). Samples were then filled in straws (CryoBioSytems IMV, France) by an automatic aliquoting system (DIVA, CryoBioSytems IMV, France). To minimize freeze–thaw cycles, samples were sorted in a cryogenic work bench (temperatures below − 100 °C) and automatically stored in tanks with a coolable top frame in the gas phase of liquid nitrogen (Askion, Germany) and stored for analysis^18^. Serum BDNF was assessed using an ELISA kit manufactured by R&D Systems (Wiesbaden, Germany) as previously described^4^.

### Neuroimaging—measurement of gray matter volume & thickness

T1-weighted images were acquired with a 3-T Magnetom Verio Scanner (Siemens Healthcare, Erlangen, Germany) using three-dimensional magnetization-prepared rapid gradient-echo imaging (3D MP-RAGE) protocol with the following parameters: inversion time 900 ms; repetition time 2,300 ms; echo time 2.98 ms; flip angle 9°; field of view 256 × 240 × 176 mm; voxel size 1 × 1 × 1 mm. To analyze gray matter volume and cortical thickness, T1-weighted images were preprocessed using FreeSurfer version 5.3.0 (https://surfer.nmr.mgh.harvard.edu/)^19^.

MR images were preprocessed using the standard pipeline recon-all. After normalization and skull-stripping of the T1-weighted images, cortical tissue boundaries were reconstructed and transformed to a subject-specific surface mesh. The distance between pial and gray/white matter surfaces at each vertex location of the mesh was calculated in order to obtain cortical thickness measurements^20^. Based on Desikan-Killiany’s cortical parcellation, regional cortical thickness and gray matter volume was extracted separately for the several brain regions in each hemisphere and averaged for the analysis. All images were visually checked for misplaced tissue boundaries and manually corrected if necessary.

### Statistics

The statistical analysis was performed in SPSS Version 24 (IBM Corp., Armonk, NY, USA). After the visual assessment of data distributions, gray matter volume, normalized to total intracranial volume (TIV), and cortical thickness estimates were correlated with sBDNF levels by calculating Pearson’s correlation coefficients separately for each group. First, we used the uncorrected p value < 0.05 (one-tailed, directed hypothesis). We subsequently corrected for multiple comparisons using the false discovery rate (FDR) approach as suggested by Benjamini–Hochberg^21^ with a threshold of 0.05. The family of tests included all segmented brain regions and mean thickness (68 regions left/right tests for the left/right analysis). We report uncorrected p values along with the calculated FDR p value^21^. These are labelled accordingly throughout the tables in bold. Interaction effects were tested between the significant correlations in minor depression and healthy control groups by using Fisher’s z-test. Subgroup analysis was performed post hoc according to the same procedures as the main analysis. Figures were prepared by MP in Blender 2.78 software (https://www.blender.org/) using the Desikan-Killiani template by Prof. Anderson Winkler (https://brainder.org/research/brain-for-blender/).

## Results

### Participants’ characteristics

Subjects with minor depressive episode were not significantly different from control subjects in terms of age, sex, body mass index (BMI), and amount of white matter hyperintensities as rated using the Fazekas scale. Levels of sBDNF were also comparable, i.e. not significantly different, between both groups (see Table 1).

**Table 1:** Participants’ characteristics.

	Subjects with minor depression	Healthy subjects	p-value
N (with history of depression)	20 (12)	40	–
Sex (male/female)	5/15	10/30	1.0
Age (years)	70.3 (4.3)	69.6 (4.3)	0.57
Fazekas score (0/1/2)	6/12/2	12/23/5	0.90
BMI (kg/m^2^)	28.3 (5.2)	28.4 (5.2)	0.91
sBDNF (µg/l)	26.0 (5.1)	25.7 (7.1)	0.84

Chi-square test for sex, independent sample t test for age, body mass index (BMI), serum brain derived neurotrophic factor (sBDNF), Mann–Whitney U test for the Fazekas score.

### Cortical thickness

**Figure 1:**
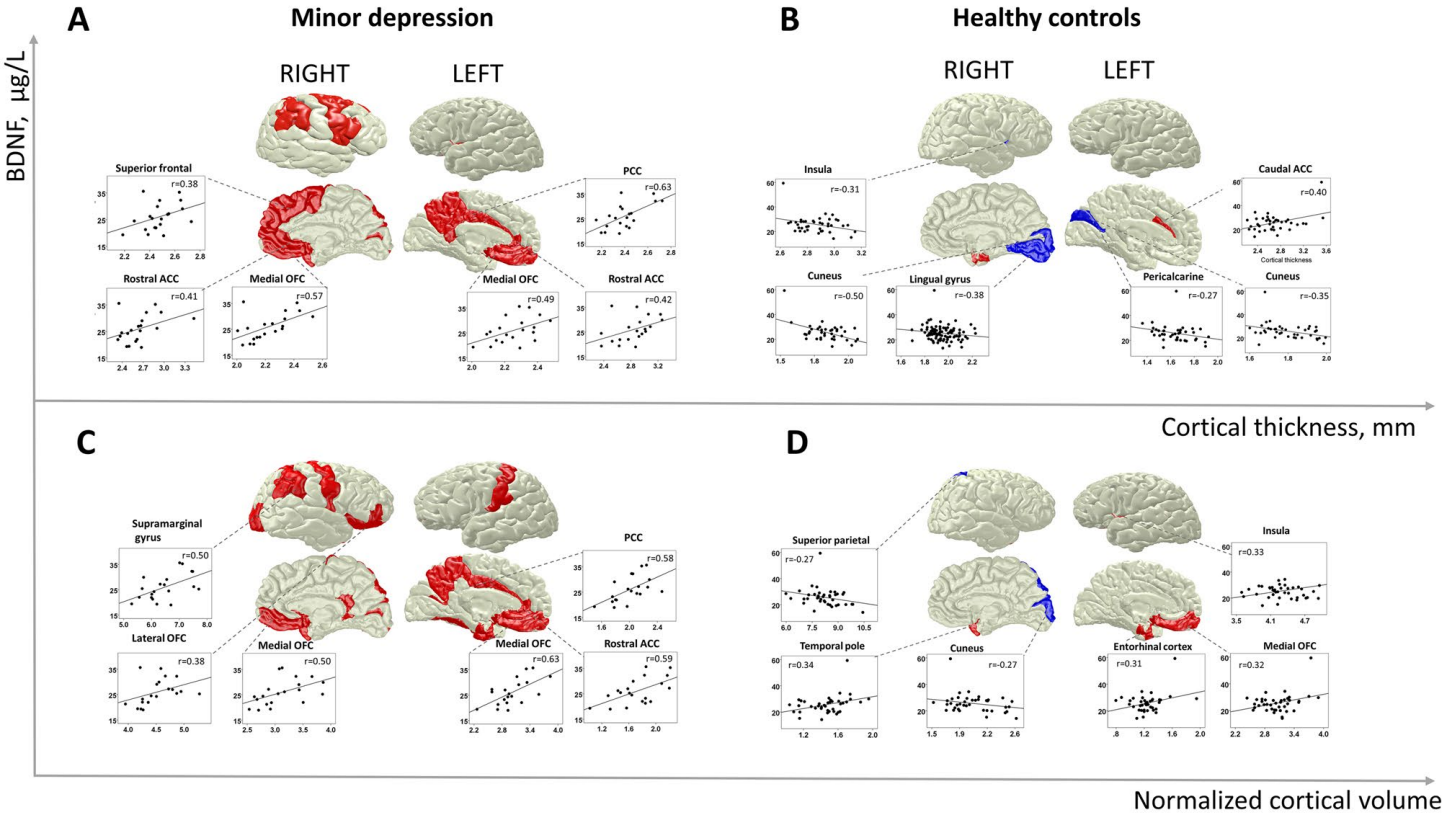
Correlation of serum BDNF with cortical thickness and normalized cortical volume in subjects with minor depression and healthy controls. (**A**) Correlation of sBDNF with cortical thickness in subjects with minor depression; (**B**) Correlation of sBDNF with cortical thickness in healthy controls; (**C**) Correlation of sBDNF with cortical volume normalized to total intracranial volume in subjects with minor depression; (**D**) Correlation of sBDNF with cortical volume normalized to total intracranial volume in healthy controls; *BDNF* Brain-Derived Neurotrophic Factor, *ACC* anterior cingulate cortex, *OFC* orbitofrontal cortex, *PCC* posterior cingulate cortex. Figures were prepared in Blender 2.78 software (https://www.blender.org/) using the Desikan-Killiani template by Anderson Winkler (https://brainder.org/research/brain-for-blender/).

Cortical thickness, and gray matter volume, were not statistically different between both groups (Supplementary Table 1 and 2), whereas sBDNF correlated with imaging parameters. At p < 0.05, we observed a positive correlation between sBDNF and cortical thickness only in the minor depression group as illustrated in Fig. 1 and Table 2. On the uncorrected level, sBDNF positively correlated with cortical thickness in the left medial orbitofrontal, the rostral and caudal anterior cingulate cortex, posterior and isthmus cingulate cortex, and the insula and precuneus. In the right hemisphere we observed positive correlations between sBDNF and cortical thickness in the medial orbitofrontal, superior frontal, rostral anterior cingulate cortex, superior parietal cortex, temporal pole and transverse temporal, as well as with the supramarginal, postcentral and pericalcarine gyrus (Fig. 1). No regions remained significant after the FDR correction for multiple comparisons p_FDR_ < 0.05 (see Table 2).

**Table 2:** Correlation between cortical thickness and serum BDNF in subjects with minor depression and healthy controls.

Region of interest	Subjects with minor depression			Healthy controls				Interaction analysis	
	Pearson’s correlation	p-value	pFDR 0.05	Pearson’s correlation	p-value		pFDR 0.05	Fisher’s z	p-value
**Left hemisphere**									
Bank of the superior temporal sulcus	− 0.21	0.18	0.03	− 0.03	0.44		0.04	–	–
Caudal anterior cingulate	**0.39**	**0.05**	**0.01**	**0.40**	**0.01**		**0.001**	− **0.04**	**0.48**
Caudal middle frontal	0.16	0.25	0.04	− 0.03	0.44		0.04	–	–
Cuneus	0.24	0.16	0.03	− **0.35**	**0.01**		**0.004**	**2.08**	**0.02**
Entorhinal cortex	0.28	0.12	0.03	0.17	0.14		0.02	–	–
Frontal pole	0.11	0.33	0.04	0.14	0.19		0.02	–	–
Fusiform gyrus	0.34	0.07	0.02	0.19	0.13		0.01	–	–
Inferior parietal gyrus	0.03	0.45	0.05	0.25	0.06		0.01	–	–
Inferior temporal gyrus	0.35	0.06	0.01	0.23	0.08		0.01	–	–
Insula	**0.40**	**0.04**	**0.01**	− 0.14	0.20		0.02	**1.90**	**0.03**
Isthmus cingulate	**0.49**	**0.01**	**0.004**	− 0.04	0.40		0.03	**1.96**	**0.03**
Lateral occipital sulcus	− 0.04	0.44	0.05	0.01	0.49		0.05	–	–
Lateral orbitofrontal cortex	0.24	0.15	0.03	0.03	0.44		0.04	–	–
Lingual gyrus	0.18	0.22	0.04	− 0.23	0.07		0.01	–	–
Medial orbitofrontal cortex	**0.49**	**0.01**	**0.003**	0.19	0.12		0.01	1.16	0.12
Middle temporal gyrus	0.12	0.31	0.04	0.23	0.08		0.01	–	–
Paracentral gyrus	0.16	0.25	0.04	− 0.02	0.46		0.04	–	–
Parahippocampal gyrus	− 0.04	0.44	0.05	0.13	0.21		0.02	–	–
Pars opercularis	0.31	0.09	0.02	0.02	0.45		0.04	–	–
Pars orbitalis	− 0.04	0.44	0.05	− 0.001	0.50		0.05	–	–
Pars triangularis	0.04	0.44	0.05	− 0.24	0.07		0.01	–	–
Pericalcarine cortex	0.35	0.07	0.02	− **0.27**	**0.05**		**0.01**	2.17	0.15
Postcentral gyrus	0.13	0.29	0.04	− 0.20	0.11		0.01	–	–
Posterior cingulate cortex	**0.63**	**0.002**	**0.001**	0.18	0.14		0.01	**1.90**	**0.03**
Precentral gyrus	0.10	0.34	0.04	0.07	0.34		0.03	–	–
Precuneus	**0.46**	**0.02**	**0.01**	− 0.03	0.43		0.04	**1.69**	**0.05**
Rostral anterior cingulate cortex	**0.42**	**0.03**	**0.01**	0.04	0.41		0.04	1.39	0.82
Rostral middle frontal cortex	0.32	0.08	0.02	0.14	0.19		0.02	–	–
Superior frontal gyrus	0.32	0.09	0.02	0.03	0.44		0.04	–	–
Superior parietal gyrus	− 0.16	0.25	0.04	− 0.02	0.44		0.04	–	–
Superior temporal gyrus	0.25	0.15	0.03	0.09	0.30		0.03	–	–
Supramarginal gyrus	0.17	0.24	0.04	− 0.04	0.41		0.04	–	–
Temporal pole	0.18	0.22	0.03	0.24	0.07		0.01	–	–
Transverse temporal gyrus	0.25	0.15	0.03	0.14	0.20		0.02	–	–
**Right hemisphere**									
Banks of the superior temporal sulcus	0.32	0.09	0.02	− 0.01	0.48		0.05	–	–
Caudal anterior cingulate	0.15	0.26	0.04	0.09	0.29		0.03	–	–
Caudal middle frontal	**0.49**	**0.02**	**0.01**	0.08	0.31		0.03	1.54	0.62
Cuneus	0.26	0.13	0.03	− **0.50**	**0.001**		**0.001**	**2.79**	** < 0.001**
Entorhinal cortex	0.29	0.11	0.03	**0.36**	**0.01**		**0.003**	− 0.26	0.40
Frontal pole	− 0.03	0.45	0.05	0.17	0.14		0.02	–	–
Fusiform gyrus	0.30	0.10	0.02	− 0.17	0.15		0.02	–	–
Inferior parietal gyrus	0.29	0.11	0.03	0.19	0.12		0.01	–	–
Inferior temporal gyrus	0.30	0.10	0.02	0.20	0.11		0.01	–	–
Insula	0.32	0.08	0.02	− **0.31**	**0.03**		**0.004**	**2.24**	**0.01**
Isthmus cingulate	0.10	0.34	0.04	0.06	0.37		0.03	–	–
Lateral occipital sulcus	0.34	0.07	0.02	0.02	0.46		0.04	–	–
Lateral orbitofrontal cortex	0.13	0.29	0.04	0.10	0.26		0.03	–	–
Lingual gyrus	0.33	0.08	0.02	− **0.38**	**0.01**		**0.002**	**2.53**	**0.01**
Medial orbitofrontal cortex	**0.57**	**0.005**	**0.001**	− 0.13	0.21		0.02	**2.65**	** < 0.001**
Middle temporal gyrus	0.34	0.07	0.02	0.14	0.19		0.02	–	–
Paracentral gyrus	0.17	0.24	0.04	0.05	0.38		0.03	–	–
Parahippocampal gyrus	0.06	0.40	0.05	0.16	0.17		0.02	–	–
Pars opercularis	**0.48**	**0.02**	**0.01**	0.03	0.42		0.04	**1.68**	**0.05**
Pars orbitalis	0.34	0.07	0.02	0.03	0.44		0.04	–	–
Pars triangularis	0.30	0.10	0.03	0.07	0.34		0.03	–	–
Pericalcarine cortex	**0.52**	**0.01**	**0.00**	− 0.11	0.25		0.02	**2.35**	**0.01**
Postcentral gyrus	0.23	0.17	0.03	− 0.08	0.32		0.03	–	–
Posterior cingulate cortex	0.12	0.30	0.04	0.11	0.26		0.03	–	–
Precentral gyrus	**0.41**	**0.04**	**0.01**	0.01	0.47		0.05	1.44	0.07
Precuneus	0.19	0.21	0.03	− 0.12	0.24		0.02	–	–
Rostral anterior cingulate cortex	**0.41**	**0.04**	**0.01**	− 0.08	0.31		0.03	1.78	0.38
Rostral middle frontal cortex	0.22	0.18	0.03	− 0.01	0.47		0.05	–	–
Superior frontal gyrus	**0.38**	**0.05**	**0.01**	0.01	0.48		0.05	1.35	0.09
Superior parietal gyrus	**0.41**	**0.04**	**0.01**	− 0.07	0.34		0.03	**1.70**	**0.04**
Superior temporal gyrus	0.36	0.06	0.01	− 0.01	0.47		0.04	–	–
Supramarginal gyrus	**0.49**	**0.01**	**0.004**	0.01	0.47		0.05	1.78	0.38
Temporal pole	**0.44**	**0.03**	**0.01**	0.24	0.07		0.01	0.77	0.22
Transverse temporal gyrus	**0.48**	**0.02**	**0.01**	0.08		0.31	0.03	1.49	0.07

*BDNF* brain derived neurotrophic factor, 1-tailed p-values are reported, FDR p value is derived using the Benjamini–Hochberg procedure, Fisher’s z-test for interaction analysis was performed only for significant correlations. Regions significantly correlating with sBDNF at p < 0.05 are marked as bold.

In healthy subjects, contrary to our hypothesis, correlations tended to be negative (Table 2). On the uncorrected level (p < 0.05), we observed significant negative correlations between sBDNF and cortical thickness of the bilateral cuneus, right lingual gyrus, and insula. Positive correlations were observed only for the left caudal anterior cingulate cortex and right entorhinal region. Interestingly, a negative correlation between sBDNF and thickness in the right cuneus was significant at the p_FDR_ < 0.05 threshold.

Between-group interaction effects were significant for correlations between sBDNF and cortical thickness in the bilateral cuneus and insula, left medial orbitofrontal cortex, precuneus, isthmus and posterior cingulate cortex, as well as the right pericalcarine and lingual gyrus, pars opercularis and superior parietal lobule. In all these cases, we observed positive correlations in subjects with minor depression and near-zero or negative correlations in healthy participants (Table 2).

### Cortical volume

**Figure 2:**
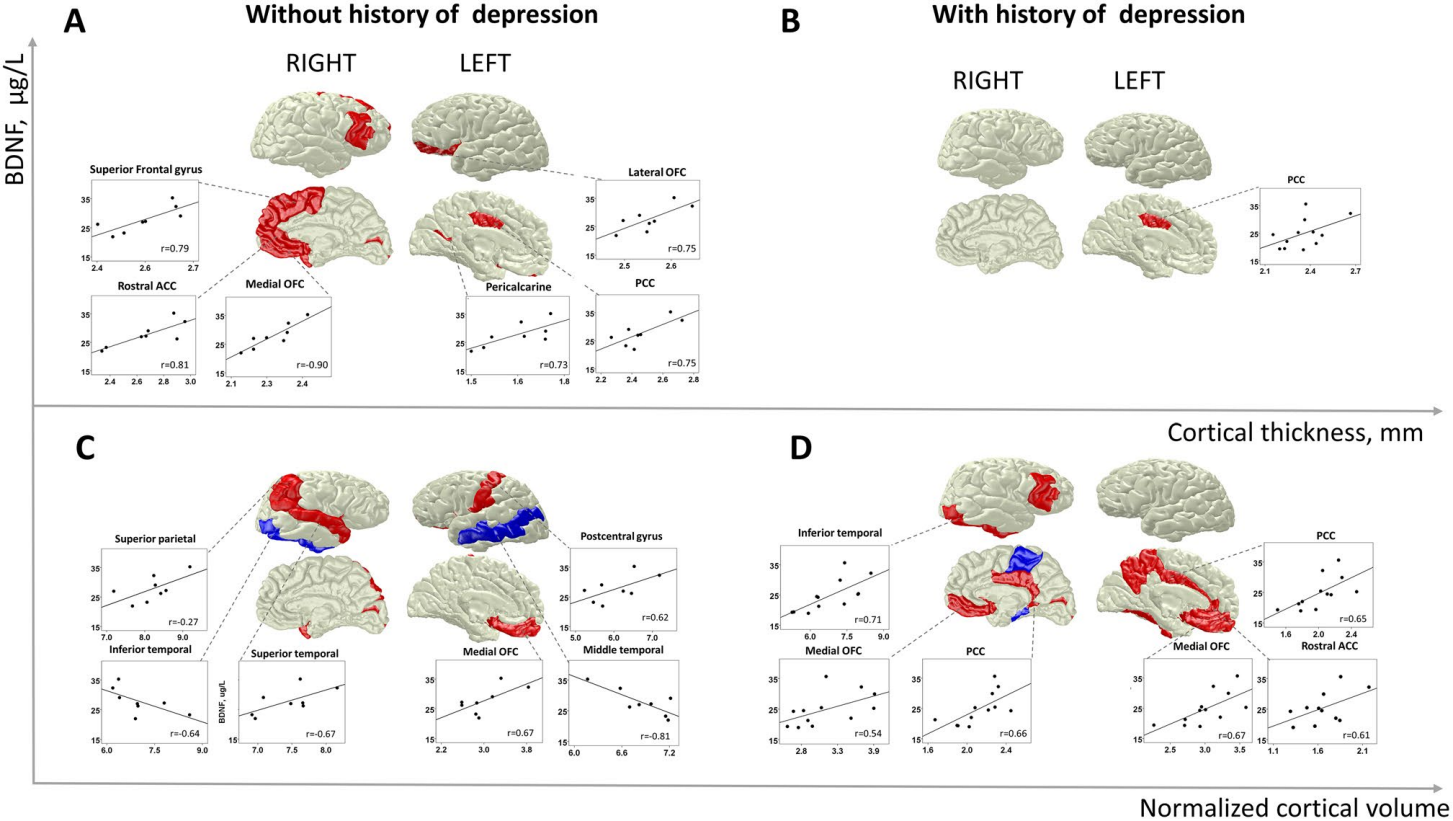
Subgroup analysis: Correlation of serum BDNF with cortical thickness and normalized cortical volume in subjects with or without history of depression. (**A**) Correlation of sBDNF with cortical thickness in subjects without history of depression; (**B**) Correlation of sBDNF with cortical thickness in subjects with history of depression; (**C**) Correlation of sBDNF with cortical volume normalized to total intracranial volume in subjects without history of depression; (**D**) Correlation of sBDNF with cortical volume normalized to total intracranial volume in subjects with history of depression; *BDNF* Brain-Derived Neurotrophic Factor, *ACC* anterior cingulate cortex, *OFC* orbitofrontal cortex, *PCC* posterior cingulate cortex. Figures were prepared in Blender 2.78 (https://www.blender.org/) using the Desikan-Killiani template (https://brainder.org/research/brain-for-blender/).

Correlations between sBDNF and volumetric data are illustrated in Fig. 2 and Table 3. The regional correlation pattern was similar between the volumetric and cortical thickness data (see Figs. 1 and 2). In subjects with minor depression at p < 0.05, sBDNF correlated positively with bilateral medial orbitofrontal and pericalcarine cortical volume. Additionally, in the left hemisphere, we observed positive correlations between sBDNF and volumes of the left rostral, caudal, and anterior cingulate, as well as the posterior cingulate cortex, precuneus, fusiform, entorhinal, and postcentral gyrus. In the right hemisphere, sBDNF positively correlated with volumes of the isthmus cingulate, lateral orbitofrontal, precentral cortex, pars orbitalis of the inferior frontal gyrus, superior parietal and superior temporal gyrus, as well as with the temporal pole and supramarginal gyrus.

**Table 3:** Correlation between normalized gray matter volume and serum BDNF in subjects with minor depression and healthy controls.

Region of Interest	Subjects with minor depression			Healthy subjects			Interaction analysis	
	Pearson’s correlation	p-value	p_FDR_ 0.05	Pearson’s correlation	p-value	p_FDR_ 0.05	Fisher’s z	p-value
**Left hemisphere**								
Banks of the superior temporal sulcus	− 0.05	0.42	0.05	0.13	0.21	0.02	–	–
Caudal anterior cingulate cortex	**0.46**	**0.02**	**0.01**	0.20	0.11	0.01	1.00	0.16
Caudal middle frontal	0.15	0.26	0.04	− 0.07	0.33	0.03	–	–
Cuneus	0.25	0.14	0.03	− 0.14	0.19	0.02	–	–
Entorhinal cortex	0.40	0.04	0.01	**0.31**	**0.02**	**0.003**	0.34	0.37
Frontal pole	0.21	0.19	0.03	0.24	0.06	0.01	–	–
Fusiform gyrus	**0.47**	**0.02**	**0.01**	0.09	0.29	0.03	1.44	0.07
Inferior parietal gyrus	0.12	0.30	0.04	0.06	0.35	0.04	–	–
Inferior temporal gyrus	0.17	0.23	0.04	0.16	0.16	0.02	–	–
Insula	0.35	0.07	0.02	0.33	0.02	0.001	0.08	0.47
Isthmus cingulate cortex	0.21	0.19	0.03	0.08	0.32	0.03	–	–
Lateral occipital sulcus	− 0.11	0.33	0.05	0.07	0.33	0.04	–	–
Lateral orbitofrontal cortex	0.30	0.10	0.02	0.16	0.16	0.01	–	–
Lingual gyrus	0.19	0.21	0.03	− 0.25	0.06	0.01	–	–
Medial orbitofrontal cortex	**0.63**	**0.001**	**0.001**	**0.32**	**0.02**	**0.001**	1.39	0.08
Middle temporal gyrus	0.13	0.29	0.04	0.12	0.24	0.03	–	–
Paracentral gyrus	− 0.23	0.16	0.03	− 0.09	0.29	0.03	–	–
Parahippocampal gyrus	0.11	0.32	0.05	0.21	0.10	0.01	–	–
Pars opercularis	0.27	0.12	0.02	− 0.07	0.33	0.04	–	–
Pars orbitalis	0.15	0.27	0.04	0.13	0.20	0.02	–	–
Pars triangularis	0.14	0.27	0.04	− 0.15	0.17	0.02	–	–
Pericalcarine cortex	**0.40**	**0.04**	**0.01**	− 0.14	0.20	0.02	**1.9**	**0.03**
Postcentral gyrus	**0.45**	**0.02**	**0.01**	− **0.26**	**0.05**	**0.01**	**2.6**	**0.005**
Posterior cingulate cortex	**0.58**	**0.004**	**0.002**	− 0.02	0.46	0.05	**2.3**	**0.01**
Precentral gyrus	− 0.01	0.48	0.05	0.02	0.44	0.05	–	–
Precuneus	**0.57**	**0.004**	**0.003**	− 0.11	0.25	0.03	**2.57**	**0.005**
Rostral anterior cingulate cortex	**0.59**	**0.003**	**0.001**	0.13	0.21	0.02	**1.84**	**0.03**
Rostral middle frontal cortex	0.26	0.13	0.03	0.11	0.24	0.03	–	–
Superior frontal gyrus	0.09	0.36	0.05	0.05	0.37	0.04	–	–
Superior parietal gyrus	0.17	0.23	0.04	− 0.06	0.35	0.04	–	–
Superior temporal gyrus	0.29	0.10	0.02	0.09	0.28	0.03	–	–
Supramarginal gyrus	0.28	0.11	0.02	− 0.12	0.23	0.03	–	–
Temporal pole	0.18	0.22	0.03	0.24	0.07	0.01	–	–
Transverse temporal gyrus	0.24	0.15	0.03	0.08	0.32	0.03	–	–
**Right hemisphere**								
Banks of the superior temporal sulcus	− 0.16	0.25	0.04	0.19	0.12	0.01	–	–
Caudal anterior cingulate	− 0.19	0.21	0.03	0.07	0.33	0.04	–	–
Caudal middle frontal cortex	0.28	0.12	0.02	− 0.02	0.46	0.05	–	–
Cuneus	0.09	0.35	0.05	− **0.27**	**0.05**	**0.004**	1.24	0.11
Entorhinal cortex	− 0.16	0.25	0.04	0.16	0.16	0.01	–	–
Frontal pole	0.24	0.15	0.03	− 0.03	0.42	0.05	–	–
Fusiform gyrus	0.16	0.25	0.04	− 0.23	0.08	0.01	–	–
Inferior parietal gyrus	0.14	0.28	0.04	0.06	0.35	0.04	–	–
Inferior temporal gyrus	0.34	0.07	0.02	0.23	0.08	0.01	–	–
Insula	0.30	0.10	0.02	0.14	0.19	0.02	–	–
Isthmus cingulate cortex	**0.42**	**0.03**	**0.01**	− 0.02	0.45	0.05	**1.6**	**0.05**
Lateral occipital gyrus	**0.37**	**0.05**	**0.01**	0.05	0.37	0.04	1.15	0.12
Lateral orbitofrontal sulcus	**0.38**	**0.05**	**0.01**	0.12	0.23	0.03	0.95	0.17
Lingual gyrus	0.34	0.07	0.02	− 0.24	0.07	0.01	–	–
Medial orbitofrontal cortex	**0.50**	**0.01**	**0.004**	− **0.05**	0.38	0.04	**2.03**	**0.02**
Middle temporal gyrus	0.22	0.18	0.03	− 0.07	0.34	0.04	–	–
Paracentral gyrus	− 0.22	0.17	0.03	0.12	0.23	0.03	–	–
Parahippocampal gyrus	− 0.14	0.28	0.04	0.17	0.15	0.01	–	–
Pars opercularis	0.31	0.09	0.02	0.01	0.47	0.05	–	–
Pars orbitalis	**0.39**	**0.04**	**0.01**	0.11	0.25	0.03	1.03	0.15
Pars triangularis	0.26	0.14	0.03	0.15	0.19	0.02	–	–
Pericalcarine cortex	**0.40**	**0.04**	**0.01**	− 0.04	0.40	0.04	1.59	0.06
Postcentral gyrus	− 0.003	0.50	0.05	− 0.14	0.20	0.02	–	–
Posterior cingulate cortex	0.34	0.07	0.02	0.06	0.35	0.04	–	–
Precentral gyrus	**0.48**	**0.02**	**0.01**	0.07	0.34	0.04	1.55	0.06
Precuneus	0.36	0.06	0.02	− 0.06	0.36	0.04	–	–
Rostral anterior cingulate cortex	− 0.21	0.19	0.03	0.13	0.21	0.02	–	–
Rostral middle frontal cortex	0.16	0.25	0.04	− 0.14	0.19	0.02	–	–
Superior frontal gyrus	0.17	0.23	0.04	0.19	0.11	0.01	–	–
Superior parietal gyrus	**0.39**	**0.04**	**0.01**	− **0.27**	**0.05**	**0.004**	**2.34**	**0.01**
Superior temporal gyrus	0.42	0.03	0.01	− 0.09	0.29	0.03	–	–
Supramarginal gyrus	**0.55**	**0.01**	**0.004**	− **0.15**	0.18	0.02	**2.6**	**0.004**
Temporal pole	**0.49**	**0.02**	**0.01**	**0.34**	**0.02**	**0.001**	0.6	0.27
Transverse temporal gyrus	0.35	0.07	0.02	0.01	0.48	0.05	–	–

*BDNF* brain derived neurotrophic factor, 1-tailed p values are reported, FDR p value is derived using the Benjamini–Hochberg procedure, Fisher’s z-test for interaction analysis was performed only for significant correlations. Regions significantly correlating with sBDNF at p = 0.05 are marked as bold.

In healthy subjects, negative correlations at p < 0.05 were found between sBDNF and volumes of the right superior parietal cortex, right cuneus, lingual and fusiform, as well as with the left postcentral, and lingual gyrus. None of these correlations remained significant after FDR correction.

Interaction effects were significant for correlations of sBDNF with volumes of the left posterior and rostral anterior cingulate cortex, precuneus, postcentral, lingual gyrus, as well as for correlations with right medial orbitofrontal, middle temporal, lingual, superior parietal, superior temporal and supramarginal volumes. Similar to cortical thickness, positive correlations characterized the minor depression group, and negative ones the healthy control group.

### Subgroup analysis

Finally, we performed a post hoc subgroup analysis to investigate potential differences between persons with and without a history of depression (n = 8 vs n = 12). The results are depicted in Supplementary Tables 3–6 and Fig. 2. Interestingly, cortical thickness was larger in subjects without history of depression (Supplementary Table 3).

Further analysis showed that correlation between cortical thickness and sBDNF in the minor depression group was driven by subjects without a history of depression. Correlation between sBDNF and right medial orbitofrontal cortical thickness in this subgroup remained significant after FDR correction. Interaction effects between both subgroups were significant for the left lateral orbitofrontal gyrus, right medial orbitofrontal gyrus, right pars triangularis of the inferior frontal gyrus, the rostral anterior cingulate cortex, and superior frontal gyrus. In all regions, correlations in subjects with first-episode minor depression were significantly higher than in subjects with recurrent depression.

Gray matter volume correlated both positively and negatively with sBDNF in both subgroups. However, none of these correlations remained significant after FDR correction. Interaction effects were significant for correlation between sBDNF and left middle temporal, right pericalcarine, and right posterior cingulate volumes. In all these cases, negative correlations were observed in subjects with first-episode minor depression and positive correlations in subjects with recurrent episode.

## Discussion

To our knowledge, this is the first structural MRI study investigating the correlation between sBDNF and gray matter parameters in minor depression. At the uncorrected level (p < 0.05) positive correlation was detected in multiple depression-related regions in subjects with minor depressive episode, but not in the control group. The respective interaction effects were significant. The post hoc analysis revealed that correlations with cortical thickness were driven by subjects with first-episode minor depression, while volumetric data showed mixed effects. Though most of these correlations remained non-significant after the FDR correction, they should inform future studies about the effect direction, effect size, and required sample size.

### Imaging phenotype matters—cortical thickness should be preferred to cortical volume in depression

Following a recent publication from the field of imaging genetics^17^, it is reasonable to argue that thickness and volume estimates are not interchangeable also in clinical investigations. In the FreeSurfer estimations gray matter volume is a product of cortical area by cortical thickness^16,17^. Since cortical area has larger inter-individual variability, volumetric measures are more influenced by the area estimates^16^. Moreover, the FreeSurfer algorithm has shown a tendency to misestimate cortical volume^22^.

Histologically, cortical area is defined by the number of neuronal columns, while cortical thickness by the number of neurons and their connections within the column^23^. The change of clinical state from euthymic to depressed is unlikely to alter the number of neuronal columns, and, therefore, cortical area and volume. Furthermore, sBDNF is a dynamic measure^24,25^. In light of the neurotrophic hypothesis, a number of neuronal connections is thought to decrease due to deficiency of neurotrophic factors in depression^6^. Therefore, we suggest that cortical thickness is much more useful for clinical studies compared to cortical volume to examine state changes in depression. Accordingly, we will further discuss results for this parameter only.

### Correlation between serum BDNF and regional cortical thickness seems to be relevant in early minor depressive states

In this study, sBDNF correlated positively with cortical thickness of numerous brain regions in minor depression. Though none of these correlations remained significant after the rigorous FDR correction, the total number of correlations was substantially higher than the expected at 5% false-positive rate (3.4 significant results are expected out of 68). Moreover, note that correlation coefficients reached relatively high values, explaining a high amount of variability in the data. sBDNF correlated positively with the thickness of the bilateral medial orbitofrontal cortex and rostral anterior cingulate, left cingulate cortex, insula, and right superior frontal gyrus. These regions are typically activated in functional MRI paradigms that assess emotion regulation in healthy subjects^26,27^ and in major depression^22,27,28^, and show changes in structure and glucose metabolism in MDD as revealed by systematic and quantitative meta-analyses^29^ and histopathological studies with glial and later neuronal alterations^30–32^.

Whether this correlation is specific to minor depression as compared to major depression remains to be investigated. Some considerations may be drawn from other studies of cortical thickness and sBDNF. Cortical thinning was robustly detected in patients with first episode major depression in a large scale study of ENIGMA consortium^33^, as well as smaller studies^34–36^. In minor depression we did not observe these effects^37^. Serum BDNF has been unchanged in first episode major depression^38^ and in minor depression^4^. One study has reported a positive correlation between sBDNF and hippocampal volume in first episode major depression in a region-of-interest analysis^39^.

An earlier study, investigating the relation of sBDNF to cortical thickness in patients with recurrent major depression, did not show such a correlation^14^. These patients had a recurrent severe (major) depressive disorder, which likely exhausted BDNF resources. Our previous meta-analysis investigating the effects of electro-convulsive therapy on BDNF in such patients showed no response of sBDNF to therapy^40^. In patients with less severe depressive disorder sBDNF responds much better to anti-depressive treatment^9^. In line with this argument, our minor depression subtype analyses revealed that the correlation between sBDNF and cortical thickness was driven by subjects without a history of depression. In summary, a significant positive correlation between sBDNF and cortical thickness might be only relevant in early depressive states and might indicate a compensatory mechanism, because it was neither detected in healthy controls nor in minor depressive states with a history of depression. With respect to healthy controls, we replicated previous findings^14^.

Regions correlating with sBDNF in our study substantially overlapped with regional cortical thinning in MDD shown in a recent very powerful meta-analysis^33^. Here, the bilateral medial orbitofrontal cortex, fusiform gyrus, insula, rostral anterior and posterior cingulate cortex and, unilaterally, the left middle temporal gyrus, right inferior temporal gyrus, and right caudal anterior cingulate cortex were significantly thinner in the MDD group than in healthy controls. The obtained effect sizes for cortical thinning were relatively small in this meta-analysis (Cohen’s d − 0.13 to 0.49)^33^. This suggests that large sample sizes are required for such studies.

We have shown here for the first time that in minor depression the correlation of sBDNF with cortical thickness is significantly different from controls. The subtype analysis suggested that this correlation was mainly driven by subjects with first episode depression. These data provide insight into the early mechanisms of depression with a focus on neuroendocrine mechanisms, possibly indicating an early compensatory mechanism, similar to other diseases^41,42^. Furthermore, it also shows that no universal positive correlation between brain measures and BDNF exists. Similarly, animal studies have shown that correlations between brain BDNF and sBDNF is very much region- and strain-specific^40^.

Whilst cortical thickness is a relatively straightforward measure, biological processes, reflected by sBDNF, are less understood. It has been long supposed that sBDNF reflects cortical and hippocampal secretion of BDNF^43,44^. However, a recent study has shown that sBDNF is instead derived from megakaryocytes^45^ and not from the brain. Therefore, mechanisms linking brain and serum BDNF are yet to be further examined.

Both cortical thickness^46^ and sBDNF^9,47^ are reduced in MDD. In our previous reports comparing subjects with minor depression, we found neither sBDNF differences^4^ nor differences in cortical measures^37^. The evidence we provide here is correlational and by no means causative. However, we might have observed an early sign of neurotrophic function in early subclinical depression, not yet visible on the biomarker or whole-brain level. This observation should be confirmed by future studies.

## Limitations

Our study has a number of limitations. Firstly, due to the unexpectedly low prevalence of minor depression in our sample, which originated from a large population-based study with approximately 2,500 participants, our sample size was relatively small and we had to include subjects with and without a history of depression. Because our study is the first one in minor depression, an a priori power analysis was not feasible. A previous study investigating the correlation between sBDNF and hippocampal volumes in early major depression used a comparable sample size n = 25^39^, suggesting we had enough statistical power. To increase power, we matched our sample on a 1:2 basis to healthy controls. Secondly, we did not have precise information on the duration of minor depressive state burden, which might be an additional parameter of interest for further analyses. Although the ELISA kits used for sBDNF quantification were not optimal according to a recent publication^48^, these kits were purchased prior to this publication. We used a whole-brain approach guaranteeing data-driven statistics in both cortical thickness and volume. Although only a minority of results survived correction for multiple comparisons using the FDR procedure, we underlined validity of our findings by interaction analyses demonstrating specificity compared to healthy subjects. Future studies are necessary to prove our pilot findings in larger and preferably multi-centric cohorts. Finally, we did not use the voxel-wise estimation, because we wanted to make our data comparable to the recent meta-analysis by the ENIGMA consortium.

## Summary

In this study, we observed a positive correlation between serum BDNF measurements and structural gray matter estimates in minor depression. The correlation between sBDNF and imaging parameters was region- and condition-dependent. These findings require verification in larger samples considering a-priori power estimations and controlling for the duration of depression burden. Furthermore, our analysis suggests that cortical thickness is a more suitable structural parameter for biomarker studies than gray matter volume, at least in studies of depression.

## Supplementary information

Supplementary file1
