# Supplementary material for: Serum BDNF levels correlate with regional cortical thickness in minor depression: a pilot study
Source: Sci Rep. 2020 Sep 3;10:14524. doi: 10.1038/s41598-020-71317-y (PMC7471294; doi:10.1038/s41598-020-71317-y)
Supplement: Supplementary file 1 — Supplementary file1 [file 41598_2020_71317_MOESM1_ESM.docx]

**Title: Serum BDNF levels correlate with regional cortical thickness in minor depression: a pilot study.**

M. Polyakova^1,2,3,9^ , F. Beyer^1,10^, K. Mueller^1^, C. Sander^2,3^,V. Witte^1,3^, L. Lampe^1,3^, F. Rodrigues^3,4^, S. Riedel-Heller^3,4^, J. Kratzsch^3.5^ K. T. Hoffmann^6^. A. Villringer^1,3,7^, P. Schoenknecht^2,3,8^, M. L. Schroeter^1,3,9^.

**Supplementary Table 1. Comparison of cortical thickness between minor depression and healthy control groups.**

| Cortical regions | Minor depression  Mean (SD) | Healthy subjects  Mean (SD) | t | Significance  (2-tailed) |
| --- | --- | --- | --- | --- |
| Left hemisphere | | | | |
| Bank of superior temporal sulcus | 2.3 (0.2) | 2.4 (0.1) | -1.3 | 0.2 |
| Caudal anterior cingulate | 2.7 (0.2) | 2.7 (0.3) | 0.3 | 0.8 |
| Caudal middle frontal | 2.4 (0.1) | 2.4 (0.1) | -0.9 | 0.4 |
| Cuneus | 1.8 (0.1) | 1.8 (0.1) | -1.0 | 0.3 |
| Entorhinal | 3.3 (0.3) | 3.4 (0.3) | -0.4 | 0.7 |
| Frontal pole | 2.6 (0.3) | 2.6 (0.2) | -1.7 | 0.1 |
| Fusiform | 2.6 (0.1) | 2.6 (0.1) | 0.6 | 0.6 |
| Inferior parietal | 2.3 (0.1) | 2.3 (0.1) | 0.1 | 0.9 |
| Inferior temporal | 2.6 (0.2) | 2.6 (0.1) | -0.1 | 0.9 |
| Isthmus cingulate | 2.3 (0.2) | 2.3 (0.2) | 0.3 | 0.8 |
| Lateral occipital | 2.1 (0.1) | 2.1 (0.1) | -1.5 | 0.2 |
| Lateral orbitofrontal | 2.5 (0.1) | 2.5 (0.1) | -0.2 | 0.9 |
| Lingual | 1.9 (0.1) | 1.9 (0.1) | 0.3 | 0.8 |
| Medial orbitofrontal | 2.3 (0.1) | 2.3 (0.1) | -0.8 | 0.4 |
| Middle temporal | 2.7 (0.1) | 2.7 (0.1) | -0.7 | 0.5 |
| Parahippocampal | 2.7 (0.3) | 2.7 (0.4) | -0.8 | 0.4 |
| Paracentral | 2.3 (0.1) | 2.3 (0.1) | -0.7 | 0.5 |
| Pars opercularis | 2.4 (0.2) | 2.4 (0.1) | -1.8 | 0.1 |
| Pars orbitalis | 2.6 (0.2) | 2.6 (0.2) | -0.5 | 0.6 |
| Pars triangularis | 2.3 (0.1) | 2.3 (0.1) | -0.3 | 0.8 |
| Pericalcarine | 1.6 (0.1) | 1.6 (0.1) | -0.1 | 0.9 |
| Postcentral | 2.0 (0.1) | 2.0 (0.1) | -0.8 | 0.4 |
| Posterior cingulate | 2.4 (0.2) | 2.4 (0.1) | 0.2 | 0.9 |
| Precentral | 2.4 (0.2) | 2.4 (0.1) | -1.0 | 0.3 |
| Precuneus | 2.2 (0.1) | 2.2 (0.1) | -1.2 | 0.2 |
| Rostral anterior cingulate | 2.8 (0.3) | 2.8 (0.2) | 0.4 | 0.7 |
| Rostral middle frontal | 2.2 (0.1) | 2.2 (0.1) | -0.9 | 0.4 |
| Superior frontal | 2.5 (0.2) | 2.5 (0.1) | -0.1 | 1.0 |
| Superior parietal | 2.1 (0.1) | 2.1 (0.1) | -1.0 | 0.3 |
| Superior temporal | 2.6 (0.2) | 2.6 (0.1) | -0.1 | 0.9 |
| Supramarginal | 2.4 (0.1) | 2.4 (0.1) | -1.8 | 0.1 |
| Temporal pole | 3.5 (0.3) | 3.5 (0.3) | -0.2 | 0.8 |
| Transverse temporal | 2.3 (0.2) | 2.2 (0.1) | -0.3 | 0.8 |
| Insula | 2.8 (0.2) | 2.9 (0.1) | 0.3 | 0.8 |
| Mean thickness | 2.3 (0.1) | 2.4 (0.1) | -0.9 | 0.4 |
| Right hemisphere | | | | |
| Bank of superior temporal sulcus | 2.5 (0.1) | 2.5 (0.2) | 0.1 | 1.0 |
| Caudal anterior cingulate | 2.4 (0.2) | 2.4 (0.2) | 1.0 | 0.3 |
| Caudal middle frontal | 2.4 (0.1) | 2.4 (0.1) | -0.6 | 0.6 |
| Cuneus | 1.8 (0.1) | 1.8 (0.1) | -0.2 | 0.8 |
| Entorhinal | 3.5 (0.4) | 3.5 (0.4) | 0.4 | 0.7 |
| Frontal pole | 2.5 (0.3) | 2.5 (0.2) | 0.3 | 0.7 |
| Fusiform | 2.7 (0.2) | 2.7 (0.1) | 0.9 | 0.4 |
| Inferior parietal | 2.4 (0.1) | 2.4 (0.1) | -1.0 | 0.3 |
| Inferior temporal | 2.7 (0.1) | 2.7 (0.1) | 1.1 | 0.3 |
| Isthmus cingulate | 2.2 (0.2) | 2.2 (0.2) | 0.6 | 0.6 |
| Lateral occipital | 2.2 (0.2) | 2.2 (0.1) | 0.4 | 0.7 |
| Lateral orbitofrontal | 2.5 (0.1) | 2.5 (0.1) | 0.5 | 0.6 |
| Lingual | 2.0 (0.1) | 2.0 (0.1) | 1.0 | 0.4 |
| Medial orbitofrontal | 2.2 (0.1) | 2.2 (0.1) | -0.5 | 0.6 |
| Middle temporal | 2.8 (0.1) | 2.8 (0.1) | -0.2 | 0.9 |
| Parahippocampal | 2.7 (0.3) | 2.7 (0.3) | 1.1 | 0.3 |
| Paracentral | 2.3 (0.1) | 2.3 (0.1) | -1.4 | 0.2 |
| Pars opercularis | 2.4 (0.1) | 2.4 (0.1) | 0.6 | 0.5 |
| Pars orbitalis | 2.5 (0.2) | 2.5 (0.2) | 0.04 | 1.0 |
| Pars triangularis | 2.3 (0.1) | 2.3 (0.1) | -0.3 | 0.8 |
| Pericalcarine | 1.7 (0.1) | 1.7 (0.1) | 0.4 | 0.7 |
| Postcentral | 2.0 (0.1) | 2.0 (0.1) | -0. | 0.8 |
| Posterior cingulate | 2.4 (0.1) | 2.3 (0.1) | 1.0 | 0.3 |
| Precentral | 2.4 (0.2) | 2.4 (0.1) | -1.4 | 0.2 |
| Precuneus | 2.3 (0.1) | 2.3 (0.1) | -1.2 | 0.2 |
| Rostral anterior cingulate | 2.7 (0.3) | 2.7 (0.2) | -0.2 | 0.9 |
| Rostral middle frontal | 2.2 (0.1) | 2.2 (0.1) | -0.9 | 0.4 |
| Superior frontal | 2.5 (0.1) | 2.5 (0.1) | -0.1 | 0.9 |
| Superior parietal | 2.1 (0.1) | 2.1 (0.1) | -1.8 | 0.1 |
| Superior temporal | 2.7 (0.1) | 2.7 (0.1) | -0.9 | 0.4 |
| Supramarginal | 2.4 (0.1) | 2.4 (0.1) | -0.7 | 0.5 |
| Temporal pole | 3.7 (0.3) | 3.7 (0.2) | 0.2 | 0.9 |
| Transverse temporal | 2.3 (0.2) | 2.3 (0.2) | -0.6 | 0.5 |
| Insula | 2.9 (0.2) | 2.9 (0.1) | -0.02 | 1.0 |
| Mean thickness | 2.4 (0.1) | 2.4 (0.1) | -0.3 | 0.8 |

**Supplementary Table 2**

**Comparison of normalized cortical volume between the minor depression and healthy control groups.**

| Cortical regions | Minor depression  Mean (SD) | Healthy subjects  Mean (SD) | t | Significance (2-tailed) |
| --- | --- | --- | --- | --- |
| Left hemisphere | | | | |
| Bank of superior temporal sulcus | 1.6 (0.3) | 1.6 (0.3) | -0.1 | 0.9 |
| Caudal anterior cingulate | 1.3 (0.2) | 1.3 (0.3) | -0.03 | 1.0 |
| Cuneus | 1.9 (0.4) | 1.8 (0.2) | 0.4 | 0.7 |
| Entorhinal | 1.2 (0.2) | 1.2 (0.2) | -1.0 | 0.3 |
| Frontalpole | 0.5 (0.1) | 0.5 (0.1) | 0.8 | 0.4 |
| Fusiform | 6.6 (0.8) | 6.4 (0.7) | 0.9 | 0.4 |
| Inferior parietal | 7.8 (0.8) | 7.9 (0.9) | -0.2 | 0.9 |
| Inferior temporal | 7.0 (1.0) | 6.8 (0.8) | 0.9 | 0.4 |
| Insula | 4.2 (0.4) | 4.3 (0.4) | -0.7 | 0.5 |
| Isthmus cingulate | 1.7 (0.3) | 1.7 (0.2) | 0.3 | 0.8 |
| Lateral occipital | **6.9 (0.9)** | **7.5 (0.7)** | **-2.6** | **0.05** |
| Lateral orbitofrontal | 4.7 (0.4) | 4.7 (0.4) | -0.2 | 0.9 |
| Lingual | 4.3 (0.6) | 4.4 (0.6) | -0.9 | 0.4 |
| Medial orbitofrontal | 3.0 (0.4) | 3.1 (0.3) | -0.5 | 0.6 |
| Middle temporal | 6.6 (0.5) | 6.4 (0.6) | 1.4 | 0.2 |
| Paracentral | 2.1 (0.2) | 2.2 (0.3) | -1.7 | 0.1 |
| Parahippocampal | 1.5 (0.2) | 1.5 (0.2) | -1.3 | 0.2 |
| Pars opercularis | 3.0 (0.5) | 2.9 (0.4) | 0.8 | 0.4 |
| Pars orbitalis | 1.4 (0.2) | 1.3 (0.2) | 0.8 | 0.4 |
| Pars triangularis | 2.1 (0.3) | 2.1 (0.3) | -0.3 | 0.7 |
| Pericalcarine | 1.5 (0.3) | 1.5 (0.3) | -0.2 | 0.8 |
| Postcentral | 6.0 (0.7) | 6.1 (0.7) | -0.3 | 0.8 |
| Posterior cingulate | 2.0 (0.2) | 2.1 (0.3) | -1.2 | 0.2 |
| Precentral | 8.2 (1.0) | 8.5 (0.8) | -1.4 | 0.2 |
| Precuneus | 5.9 (0.4) | 6.0 (0.5) | -0.7 | 0.5 |
| Rostral anterior cingulate | 1.7 (0.3) | 1.7 (0.2) | -0.4 | 0.7 |
| Rostral middle frontal | 9.1 (1.1) | 9.3 (0.9) | -0.8 | 0.4 |
| Superior frontal | 12.7 (1.6) | 13.0 (1.0) | -1.0 | 0.3 |
| Superior parietal | 7.8 (0.6) | 8.3 (0.9) | -2.2 | 0.1 |
| Superior temporal | 7.2 (0.9) | 7.3 (0.9) | -0.7 | 0.5 |
| Supramarginal | 6.6 (0.7) | 6.8 (0.8) | -0.7 | 0.5 |
| Temporal pole | 1.7 (0.3) | 1.6 (0.2) | 0.3 | 0.7 |
| Transverse temporal | 0.8 (0.1) | 0.7 (0.1) | 0.5 | 0.7 |
| Right hemisphere | | | | |
| Bank of superior temporal sulcus | 1.5 (0.2) | 1.5 (0.3) | -0.5 | 0.6 |
| Caudal anterior cingulate | 1.4 (0.3) | 1.4 (0.2) | 0.5 | 0.6 |
| Cuneus | 1.9 (0.3) | 2.0 (0.3) | -0.8 | 0.4 |
| Entorhinal | 1.2 (0.2) | 1.2 (0.2) | -0.4 | 0.7 |
| Frontal pole | 0.7 (0.2) | 0.6 (0.1) | 0.8 | 0.4 |
| Fusiform | 6.4 (0.7) | 6.4 (0.6) | -0.4 | 0.7 |
| Inferior parietal | 9.4 (1.1) | 9.6 (1.3) | -0.5 | 0.6 |
| Inferior temporal | 6.9 (1.0) | 6.8 (0.8) | 0.6 | 0.5 |
| Isthmus cingulate | 1.5 (0.2) | 1.6 (0.2) | -0.7 | 0.5 |
| Lateral occipital | 7.1 (1.1) | 7.4 (0.8) | -1.4 | 0.2 |
| Lateral orbitofrontal | 4.5 (0.3) | 4.5 (0.4) | 0.1 | 0.9 |
| Lingual | 4.5 (0.7) | 4.5 (0.6) | 0.3 | 0.8 |
| Medial orbitofrontal | 3.1 (0.4) | 3.1 (0.3) | 0.0 | 1.1 |
| Middle temporal | 7.4 (0.7) | 7.3 (0.7) | 0.5 | 0.6 |
| Paracentral | **2.4 (0.3)** | **2.6 (0.3)** | **-2.2** | **0.03** |
| Parahippocampal | 1.5 (0.3) | 1.5 (0.3) | -0.1 | 0.9 |
| Pars opercularis | 2.4 (0.3) | 2.5 (0.3) | -0.6 | 0.5 |
| Pars orbitalis | 1.6 (0.2) | 1.6 (0.2) | -0.4 | 0.7 |
| Pars triangularis | 2.5 (0.4) | 2.5 (0.4) | 0.4 | 0.7 |
| Pericalcarine | 1.7 (0.3) | 1.6 (0.3) | 0.5 | 0.6 |
| Postcentral | 5.6 (0.6) | 5.7 (0.8) | -0.8 | 0.4 |
| Posterior cingulate | 2.1 (0.2) | 2.2 (0.3) | -0.2 | 0.8 |
| Precentral | 8.2 (0.8) | 8.4 (0.8) | -1.0 | 0.3 |
| Precuneus | 6.2 (0.5) | 6.3 (0.6) | -0.6 | 0.5 |
| Rostral anterior cingulate | 1.3 (0.2) | 1.3 (0.2) | -0.0 | 1.0 |
| Rostral middle frontal | 9.3 (0.7) | 9.6 (0.9) | -1.5 | 0.1 |
| Superior frontal | 12.6 (1.4) | 12.5 (1.0) | 0.2 | 0.9 |
| Superior parietal | 7.9 (1.0) | 8.2 (0.9) | -1.3 | 0.2 |
| Superior temporal | 7.1 (0.6) | 7.1 (0.7) | 0.1 | 1.1 |
| Supramarginal | 6.5 (0.7) | 6.3 (0.7) | 1.1 | 0.3 |
| Temporal pole | 1.5 (0.3) | 1.5 (0.2) | 0.4 | 0.7 |
| Transverse temporal | 0.6 (0.1) | 0.6 (0.1) | 0.2 | 0.8 |

**Supplementary Table 3. Subgroup analysis: Comparison of cortical thickness between subjects with and without history of depression.**

| **Cortical regions** | **Subjects without history of depression**  **(n=8)** | **Subjects with history of depression**  **(n=12)** | **t** | **Significance (2-tailed)** | |  |
| --- | --- | --- | --- | --- | --- | --- |
| **Right hemisphere** | | | | | | |
| **Bank of the superior temporal sulcus** | **2.6 (0.1)** | **2.4 (0.1)** | **3.3** | | **0.004** | |
| Caudal anterior cingulate | 2.5 (0.2) | 2.4 (0.2) | 0.1 | | 0.9 | |
| **Caudal middle frontal gyrus** | **2.5 (0.1)** | **2.3 (0.1)** | **2.8** | | **0.01** | |
| Cuneus | 1.9 (0.1) | 1.8 (0.1) | 1.5 | | 0.1 | |
| Entorhinal cortex | 3.7 (0.3) | 3.4 (0.4) | 2.0 | | 0.1 | |
| Frontal pole | 2.5 (0.2) | 2.6 (0.4) | -0.4 | | 0.7 | |
| **Fusiform gyrus** | **2.8 (0.1)** | **2.6 (0.2)** | **3.1** | | **0.01** | |
| **Inferior parietal gyrus** | **2.4 (0.1)** | **2.3 (0.1)** | **2.8** | | **0.01** | |
| **Inferior temporal gyrus** | **2.9 (0.1)** | **2.7 (0.1)** | **3.7** | | **0.002** | |
| **Insula** | **3.0 (0.2)** | **2.8 (0.1)** | **3.1** | | **0.01** | |
| Isthmus cingulate | 2.3 (0.1) | 2.2 (0.2) | 1.0 | | 0.3 | |
| Lateral occipital sulcus | 2.3 (0.1) | 2.2 (0.2) | 1.6 | | 0.1 | |
| Lateral orbitofrontal cortex | 2.5 (0.1) | 2.4 (0.2) | 1.4 | | 0.1 | |
| **Lingual gyrus** | **2.1 (0.1)** | **1.9 (0.1)** | **2.9** | | **0.01** | |
| Medial orbitofrontal cortex | 2.3 (0.1) | 2.2 (0.2) | 1.4 | | 0.2 | |
| **Middle temporal gyrus** | **2.8 (0.1)** | **2.7 (0.1)** | **2.6** | | **0.02** | |
| **Paracentral gyrus** | **2.4 (0.1)** | **2.2 (0.1)** | **3.4** | | **0.003** | |
| Parahippocampal gyrus | 2.9 (0.3) | 2.8 (0.2) | 1.0 | | 0.4 | |
| **Pars opercularis** | **2.5 (0.1)** | **2.4 (0.1)** | **3.5** | | **0.003** | |
| Pars orbitalis | 2.6 (0.2) | 2.5 (0.2) | 1.3 | | 0.2 | |
| Pars triangularis | 2.3 (0.1) | 2.2 (0.1) | 1.8 | | 0.1 | |
| Pericalcarine cortex | 1.7 (0.1) | 1.7 (0.1) | 0.3 | | 0.8 | |
| Postcentral gyrus | 2.0 (0.1) | 1.9 (0.2) | 0.4 | | 0.7 | |
| **Posterior cingulate cortex** | **2.5 (0.1)** | **2.3 (0.1)** | **2.6** | | **0.02** | |
| **Precentral gyrus** | **2.5 (0.1)** | **2.2 (0.2)** | **3.3** | | **0.004** | |
| Precuneus | 2.3 (0.1) | 2.2 (0.1) | 1.9 | | 0.1 | |
| Rostral anterior cingulate cortex | 2.7 (0.2) | 2.6 (0.3) | 0.5 | | 0.6 | |
| Rostral middle frontal cortex | 2.2 (0.1) | 2.1 (0.1) | 0.6 | | 0.5 | |
| **Superior frontal gyrus** | **2.5 (0.1)** | **2.4 (0.1)** | **2.2** | | **0.04** | |
| **Superior parietal gyrus** | **2.1 (0.1)** | **2.0 (0.1)** | **2.5** | | **0.04** | |
| **Superior temporal gyrus** | **2.7 (0.1)** | **2.6 (0.1)** | **3.2** | | **0.005** | |
| **Supramarginal gyrus** | **2.5 (0.1)** | **2.3 (0.1)** | **4.4** | | **<0.001** | |
| **Temporal pole** | **3.9(0.2)** | **3.6 (0.2)** | **3.3** | | **0.004** | |
| Transverse temporal gyrus | 2.3 (0.2) | 2.2 (0.2) | 1.1 | | 0.3 | |
| **Left hemisphere** |  |  |  | |  | |
| **Bank of the superior temporal sulcus** | **2.4 (0.2)** | **2.2 (0.1)** | **2.9** | | **0.01** | |
| Caudal anterior cingulate | 2.8 (0.3) | 2.6 (0.2) | 1.9 | | 0.1 | |
| Caudal middle frontal gyrus | 2.4(0.1) | 2.3 (0.2) | 0.7 | | 0.5 | |
| Cuneus | 1.8 (0.1) | 1.8 (0.1) | 0.5 | | 0.6 | |
| **Entorhinal cortex** | **3.5 (0.2)** | **3.2 (0.3)** | **2.5** | | **0.02** | |
| **Frontal pole** | **2.7 (0.3)** | **2.6 (0.2)** | **0.9** | | **0.4** | |
| **Fusiform gyrus** | **2.7 (0.1)** | **2.6 (0.1)** | **3.7** | | **0.002** | |
| Inferior parietal gyrus | 2.4 (0.1) | 2.3 (0.1) | 2.0 | | 0.1 | |
| **Inferior temporal gyrus** | **2.7 (0.1)** | **2.6 (0.1)** | **3.4** | | **0.003** | |
| **Insula** | **2.9 (0.1)** | **2.8 (0.2)** | **2.3** | | **0.04** | |
| Isthmus cingulate | 2.4 (0.2) | 2.3 (0.2) | 1.6 | | 0.1 | |
| Lateral occipital sulcus | 2.1 (0.1) | 2.0 (0.1) | 2.0 | | 0.1 | |
| **Lateral orbitofrontal cortex** | **2.6 (0.1)** | **2.4 (0.1)** | **2.4** | | **0.03** | |
| Lingual gyrus | 2.0 (0.1) | 1.9 (0.1) | 2.0 | | 0.1 | |
| **Medial orbitofrontal cortex** | **2.3 (0.1)** | **2.2 (0.1)** | **2.1** | | **0.05** | |
| **Middle temporal gyrus** | **2.8 (0.1)** | **2.6 (0.1)** | **4.0** | | **0.0009** | |
| Paracentral gyrus | 2.3 (0.1) | 2.2 (0.1) | 1.5 | | 0.2 | |
| **Parahippocampal gyrus** | **2.7 (0.2)** | **2.6 (0.3)** | **0.2** | | **0.86** | |
| Pars opercularis | 2.4 (0.1) | 2.3 (0.2) | 1.8 | | 0.1 | |
| **Pars orbitalis** | **2.7 (0.1)** | **2.4 (0.2)** | **3.6** | | **0.002** | |
| Pars triangularis | 2.3 (0.1) | 2.2 (0.2) | 1.6 | | 0.1 | |
| Pericalcarine cortex | 1.6 (0.1) | 1.6 (0.2) | 0.3 | | 0.8 | |
| Postcentral gyrus | 2.0 (0.1) | 2.0 (0.1) | 0.3 | | 0.8 | |
| Posterior cingulate cortex | 2.5 (0.2) | 2.4 (0.1) | 1.6 | | 0.1 | |
| Precentral gyrus | 2.5 (0.2) | 2.4 (0.2) | 1.0 | | 0.3 | |
| Precuneus | 2.2 (0.05) | 2.2 (0.1) | 1.4 | | 0.2 | |
| Rostral anterior cingulate cortex | 2.9 (0.2) | 2.8 (0.3) | 1.2 | | 0.2 | |
| **Rostral middle frontal cortex** | **2.3 (0.1)** | **2.2 (0.1)** | **2.6** | | **0.02** | |
| **Superior frontal gyrus** | **2.6 (0.1)** | **2.5 (0.2)** | **2.7** | | **0.02** | |
| Superior parietal gyrus | 2.1 (0.1) | 2.1 (0.2) | 0.8 | | 0.5 | |
| **Superior temporal gyrus** | **2.8 (0.1)** | **2.5 (0.2)** | **4.4** | | **0.0004** | |
| **Supramarginal gyrus** | **2.4 (0.1)** | **2.3 (0.1)** | **2.9** | | **0.01** | |
| **Temporal pole** | **3.7 (0.3)** | **3.4 (0.3)** | **2.6** | | **0.02** | |
| Transverse temporal gyrus | 2.3 (0.1) | 2.2 (0.2) | 1.1 | | 0.3 | |

**Supplementary Table 4. Subgroup analysis: Comparison of normalized cortical volume between subjects with and without history of depression.**

| Cortical regions | Subjects without history of depression  (n=8) | Subjects with history of depression  (n=12) | t | | Significance (2-tailed) |
| --- | --- | --- | --- | --- | --- |
| Left hemisphere |  |  | |  |  |
| Banks of the superior temporal sulcus | 1.6 (0.3) | 1.6 (0.3) | | 0.4 | 0.7 |
| Caudal anterior cingulate | 1.3 (0.2) | 1.3 (0.2) | | 0.5 | 0.6 |
| Cuneus | 1.9 (0.2) | 1.9 (0.4) | | 0.04 | 1.1 |
| Entorhinal cortex | 1.2 (0.2) | 1.2 (0.2) | | 0.5 | 0.6 |
| Frontal pole | 0.5 (0.1) | 0.5 (0.1) | | -0.1 | 0.9 |
| Fusiform gyrus | 6.7 (0.6) | 6.4 (0.9) | | 1.5 | 0.2 |
| Inferior parietal gyrus | 7.8 (0.7) | 7.8 (1.0) | | -0.1 | 0.9 |
| Inferior temporal gyrus | 7.3 (0.6) | 6.8 (1.2) | | 1.0 | 0.3 |
| Insula | 4.4 (0.3) | 4.2 (0.4) | | 1.1 | 0.3 |
| Isthmus cingulate | 1.8 (0.3) | 1.7 (0.3) | | 1.0 | 0.3 |
| Lateral occipital sulcus | 7.0 (0.4) | 6.9 (1.1) | | 0.3 | 0.8 |
| Lateral orbitofrontal cortex | 4.8 (0.2) | 4.6 (0.5) | | 1.5 | 0.2 |
| Lingual gyrus | 4.5 (0.6) | 4.2 (0.7) | | 1.2 | 0.3 |
| Medial orbitofrontal cortex | 3.0 (0.4) | 3.0 (0.4) | | -0.03 | 1.0 |
| Middle temporal gyrus | 6.8 (0.4) | 6.5 (0.6) | | 1.4 | 0.2 |
| Paracentral gyrus | 2.1 (0.3) | 2.1 (0.2) | | 0.4 | 0.7 |
| Parahippocampal gyrus | 1.5 (0.1) | 1.4 (0.2) | | 0.7 | 0.5 |
| Pars opercularis | 2.9 (0.5) | 3.1 (0.5) | | -0.5 | 0.6 |
| Pars orbitalis | 1.4 (0.1) | 1.3 (0.2) | | 1.8 | 0.1 |
| Pars triangularis | 2.1 (0.2) | 2.0 (0.3) | | 0.8 | 0.4 |
| Pericalcarine cortex | 1.4 (0.2) | 1.5 (0.3) | | -0.7 | 0.5 |
| Postcentral gyrus | 6.1 (0.7) | 6.0 (0.8) | | 0.2 | 0.8 |
| Posterior cingulate cortex | 2.0 (0.2) | 2.0 (0.3) | | -0.2 | 0.9 |
| Precentral gyrus | 8.1 (1.0) | 8.2 (1.1) | | -0.03 | 1.1 |
| Precuneus | 5.9 (0.3) | 6.0 (0.5) | | -0.6 | 0.6 |
| Rostral anterior cingulate cortex | 1.9 (0.3) | 1.6 (0.3) | | 2.1 | 0.1 |
| Rostral middle frontal cortex | 9.2 (0.7) | 9.0 (1.3) | | 0.3 | 0.8 |
| Superior frontal gyrus | 13.2 (0.4) | 12.3 (1.9) | | 1.6 | 0.1 |
| Superior parietal gyrus | 8.0 (0.4) | 7.7 (0.6) | | 1.1 | 0.3 |
| Superior temporal gyrus | **7.7 (0.7)** | **6.8 (0.9)** | | **2.2** | **0.04** |
| Supramarginal gyrus | 6.7 (0.4) | 6.6 (0.9) | | 0.1 | 0.9 |
| Temporal pole | 1.7 (0.3) | 1.6 (0.3) | | 1.1 | 0.3 |
| Transverse temporal gyrus | 0.8 (0.2) | 0.7 (0.1) | | 0.5 | 0.6 |
| Right hemisphere |  |  | |  |  |
| Banks of the superior temporal sulcus | 1.52 (0.2) | 1.5 (0.2) | | -0.3 | 0.8 |
| Caudal anterior cingulate | 1.3 (0.2) | 1.5 (0.3) | | -1.6 | 0.1 |
| Cuneus | 1.9 (0.3) | 1.9 (0.2) | | -0.2 | 0.9 |
| Entorhinal cortex | 1.1 (0.1) | 1.2 (0.2) | | -0.6 | 0.6 |
| Frontal pole | 0.7 (0.1) | 0.7 (0.2) | | -0.2 | 0.9 |
| Fusiform gyrus | 6.6 (0.6) | 6.2 (0.8) | | 1.4 | 0.2 |
| Inferior parietal gyrus | 9.5 (1.4) | 9.3 (1.0) | | 0.4 | 0.7 |
| Inferior temporal gyrus | 7.0 (0.8) | 6.8 (1.1) | | 0.5 | 0.7 |
| Insula | 4.3 (0.2) | 4.2 (0.5) | | 0.6 | 0.6 |
| Isthmus cingulate | 1.6(0.2) | 1.5 (0.2) | | 1.3 | 0.2 |
| Lateral occipital sulcus | 7.3 (0.9) | 7.0 (1.3) | | 0.6 | 0.6 |
| Lateral orbitofrontal cortex | 4.6 (0.2) | 4.5 (0.4) | | 1.0 | 0.4 |
| Lingual gyrus | 4.8 (0.4) | 4.3 (0.8) | | 1.6 | 0.1 |
| Medial orbitofrontal cortex | 3.1 (0.3) | 3.1 (0.5) | | -0.01 | 1.0 |
| Middle temporal gyrus | 7.6 (0.5) | 7.3 (0.8) | | 0.8 | 0.4 |
| Paracentral gyrus | 2.5 (0.3) | 2.4 (0.2) | | 0.8 | 0.6 |
| Parahippocampal gyrus | 1.5(0.3) | 1.5 (0.3) | | 0.3 | 0.8 |
| Pars opercularis | 2.4 (0.3) | 2.4 (0.4) | | -0.1 | 1.0 |
| Pars orbitalis | 1.7 (0.2) | 1.6 (0.2) | | 1.1 | 0.3 |
| Pars triangularis | 2.6 (0.5) | 2.5 (0.4) | | 0.3 | 0.8 |
| Pericalcarine cortex | 1.7 (0.3) | 1.7 (0.3) | | -0.1 | 0.9 |
| Postcentral gyrus | 5.6 (0.4) | 5.5 (0.7) | | 0.3 | 0.7 |
| Posterior cingulate cortex | 2.2 (0.2) | 2.1 (0.2) | | 0.4 | 0.7 |
| Precentral gyrus | 8.3 (0.6) | 8.1 (0.9) | | 0.4 | 0.7 |
| Precuneus | 6.3 (0.5) | 6.1 (0.6) | | 0.6 | 0.6 |
| Rostral anterior cingulate cortex | 1.3 (0.1) | 1.4 (0.2) | | -1.1 | 0.3 |
| Rostral middle frontal cortex | 9.3 (0.5) | 9.3 (0.8) | | -0.03 | 1.0 |
| Superior frontal gyrus | 13.1 (0.8) | 12.2 (1.6) | | 1.5 | 0.2 |
| Superior parietal gyrus | 8.2 (0.6) | 7.7 (1.1) | | 1.3 | 0.2 |
| Superior temporal gyrus | **7.4 (0.4)** | **6.9 (0.6)** | | **2.3** | **0.03** |
| Supramarginal gyrus | 6.7 (0.6) | 6.3 (0.8) | | 1.0 | 0.3 |
| Temporal pole | 1.5 (0.3) | 1.5 (0.3) | | 0.6 | 0.6 |
| Transverse temporal gyrus | 0.6 (0.2) | 0.6 (0.1) | | 0.5 | 0.6 |

**Supplementary Table 5. Subgroup analysis: Correlation between serum BDNF and cortical thickness in subjects with and without history of depression**

| Region of Interest | | Subjects without history of depression (n=8) | | | | | | Subjects with history of depression (n=12) | | | | | Interaction analysis | | |  |
| --- | --- | --- | --- | --- | --- | --- | --- | --- | --- | --- | --- | --- | --- | --- | --- | --- |
|  |  | Pearson’s correlation | | p-value | | p_FDR0_.05 | Pearson’s correlation | | | p-value | | p_FDR0_.05 | Fisher’s z | | p-value | |
| Left hemisphere | | | | | | | | | | | | | | | |  |
| Bank of the superior temporal sulcus | -0.6 | | 0.1 | | 0.01 | | | -0.4 | 0.1 | | 0.01 | | - | - | |  |
| Caudal anterior cingulate | 0.3 | | 0.2 | | 0.03 | | | 0.3 | 0.1 | | 0.01 | | - | - | |  |
| Caudal middle frontal | 0.3 | | 0.2 | | 0.03 | | | 0.1 | 0.4 | | 0.04 | | - | - | |  |
| Cuneus | 0.2 | | 0.4 | | 0.04 | | | 0.2 | 0.2 | | 0.02 | | - | - | |  |
| Entorhinal cortex | -0.1 | | 0.4 | | 0.04 | | | 0.3 | 0.2 | | 0.02 | | - | - | |  |
| Frontal pole | -0.03 | | 0.5 | | 0.05 | | | 0.1 | 0.4 | | 0.04 | | - | - | |  |
| Fusiform gyrus | -0.2 | | 0.3 | | 0.04 | | | 0.3 | 0.2 | | 0.01 | | - | - | |  |
| Inferior parietal gyrus | 0.01 | | 0.5 | | 0.05 | | | -0.2 | 0.3 | | 0.03 | | - | - | |  |
| Inferior temporal gyrus | 0.3 | | 0.3 | | 0.03 | | | 0.2 | 0.2 | | 0.02 | | - | - | |  |
| Insula | 0.4 | | 0.2 | | 0.02 | | | 0.3 | 0.2 | | 0.02 | | - | - | |  |
| Isthmus cingulate cortex | 0.4 | | 0.1 | | 0.02 | | | 0.4 | 0.1 | | 0.004 | | - | - | |  |
| Lateral occipital sulcus | 0.002 | | 0.5 | | 0.05 | | | -0.3 | 0.2 | | 0.02 | | - | - | |  |
| Lateral orbitofrontal cortex | **0.8** | | **0.02** | | **0.004** | | | 0.01 | 0.5 | | 0.05 | | **1.7** | **0.04** | |  |
| Lingual gyrus | 0.1 | | 0.4 | | 0.04 | | | 0.1 | 0.4 | | 0.04 | | - | - | |  |
| Medial orbitofrontal cortex | 0.3 | | 0.3 | | 0.03 | | | 0.5 | 0.1 | | 0.001 | | - | - | |  |
| Middle temporal gyrus | -0.6 | | 0.1 | | 0.01 | | | 0.1 | 0.4 | | 0.04 | | - | - | |  |
| Paracentral gyrus | -0.2 | | 0.3 | | 0.04 | | | 0.2 | 0.3 | | 0.03 | | - | - | |  |
| Parahippocampal gyrus | -0.3 | | 0.2 | | 0.03 | | | 0.1 | 0.4 | | 0.04 | | - | - | |  |
| Pars opercularis | -0.1 | | 0.4 | | 0.04 | | | 0.3 | 0.2 | | 0.01 | | - | - | |  |
| Pars orbitalis | -0.5 | | 0.1 | | 0.02 | | | -0.2 | 0.2 | | 0.02 | | - | - | |  |
| Pars triangularis | -0.5 | | 0.1 | | 0.02 | | | 0.1 | 0.4 | | 0.04 | | - | - | |  |
| Pericalcarine cortex | **0.7** | | **0.02** | | **0.01** | | | 0.2 | 0.3 | | 0.03 | | 1.3 | 0.1 | |  |
| Postcentral gyrus | 0.2 | | 0.4 | | 0.04 | | | 0.1 | 0.4 | | 0.04 | | - | - | |  |
| Posterior cingulate cortex | **0.8** | | **0.02** | | **0.004** | | | **0.5** | **0.05** | | **0.001** | | 0.8 | 0.2 | |  |
| Precentral gyrus | -0.1 | | 0.4 | | 0.04 | | | 0.1 | 0.4 | | 0.03 | | - | - | |  |
| Precuneus | 0.4 | | 0.2 | | 0.02 | | | 0.4 | 0.1 | | 0.003 | | - | - | |  |
| Rostral anterior cingulate cortex | 0.4 | | 0.2 | | 0.02 | | | 0.4 | 0.1 | | 0.01 | | - | - | |  |
| Rostral middle frontal cortex | 0.6 | | 0.1 | | 0.01 | | | 0.1 | 0.4 | | 0.04 | | - | - | |  |
| Superior frontal gyrus | 0.5 | | 0.1 | | 0.01 | | | 0.1 | 0.4 | | 0.04 | | - | - | |  |
| Superior parietal gyrus | -0.3 | | 0.3 | | 0.03 | | | -0.2 | 0.2 | | 0.02 | | - | - | |  |
| Superior temporal gyrus | 0.3 | | 0.2 | | 0.03 | | | -0.04 | 0.5 | | 0.04 | | - | - | |  |
| Supramarginal gyrus | -0.04 | | 0.5 | | 0.05 | | | 0.03 | 0.5 | | 0.05 | | - | - | |  |
| Temporal pole | 0.4 | | 0.2 | | 0.03 | | | -0.1 | 0.3 | | 0.03 | | - | - | |  |
| Transverse temporal gyrus | 0.2 | | 0.4 | | 0.04 | | | 0.2 | 0.3 | | 0.03 | | - | - | |  |
| Right hemisphere |  | |  | |  | | | | | | | | |  | |  |
| Bank of the superior temporal sulcus | 0.3 | | 0.2 | | 0.03 | | | 0.1 | 0.3 | | 0.03 | | - | - | |  |
| Caudal anterior cingulate | 0.5 | | 0.1 | | 0.02 | | | 0.02 | 0.5 | | 0.05 | | - | - | |  |
| Caudal middle frontal | 0.4 | | 0.2 | | 0.02 | | | 0.4 | 0.1 | | 0.01 | | - | - | |  |
| Cuneus | 0.1 | | 0.5 | | 0.05 | | | 0.3 | 0.2 | | 0.01 | | - | - | |  |
| Entorhinal cortex | 0.1 | | 0.4 | | 0.04 | | | 0.2 | 0.3 | | 0.03 | | - | - | |  |
| Frontal pole | -0.3 | | 0.3 | | 0.03 | | | 0.1 | 0.4 | | 0.04 | | - | - | |  |
| Fusiform gyrus | 0.2 | | 0.3 | | 0.04 | | | 0.2 | 0.3 | | 0.03 | | - | - | |  |
| Inferior parietal gyrus | 0.6 | | 0.1 | | 0.01 | | | 0.1 | 0.4 | | 0.04 | | - | - | |  |
| Inferior temporal gyrus | -0.4 | | 0.2 | | 0.02 | | | 0.4 | 0.1 | | 0.01 | | - | - | |  |
| Insula | 0.3 | | 0.2 | | 0.03 | | | 0.1 | 0.3 | | 0.03 | | - | - | |  |
| Isthmus cingulate cortex | -0.4 | | 0.2 | | 0.02 | | | 0.2 | 0.3 | | 0.03 | | - | - | |  |
| Lateral occipital sulcus | 0.3 | | 0.2 | | 0.03 | | | 0.3 | 0.2 | | 0.02 | | - | - | |  |
| Lateral orbitofrontal cortex | 0.3 | | 0.2 | | 0.03 | | | -0.01 | 0.5 | | 0.05 | | - | - | |  |
| Lingual gyrus | 0.1 | | 0.5 | | 0.04 | | | 0.3 | 0.2 | | 0.02 | | - | - | |  |
| Medial orbitofrontal cortex | **0.9** | | **0.001** | | **0.001** | | | 0.4 | 0.1 | | 0.01 | | **1.8** | **0.03** | |  |
| Middle temporal gyrus | 0.2 | | 0.3 | | 0.04 | | | 0.2 | 0.2 | | 0.02 | | - | - | |  |
| Paracentral gyrus | **0.6** | | **0.05** | | **0.01** | | | -0.3 | 0.2 | | 0.01 | | **1.8** | **0.03** | |  |
| Parahippocampal gyrus | 0.01 | | 0.5 | | 0.05 | | | -0.02 | 0.5 | | 0.05 | | - | - | |  |
| Pars opercularis | 0.6 | | 0.1 | | 0.01 | | | 0.3 | 0.2 | | 0.01 | | - | - | |  |
| Pars orbitalis | 0.6 | | 0.1 | | 0.01 | | | 0.1 | 0.4 | | 0.03 | | - | - | |  |
| Pars triangularis | **0.8** | | **0.01** | | **0.001** | | | -0.03 | 0.5 | | 0.05 | | **2.2** | **0.02** | |  |
| Pericalcarine cortex | **0.7** | | **0.04** | | **0.01** | | | 0.5 | 0.1 | | 0.002 | | 0.5 | 0.3 | |  |
| Postcentral gyrus | -0.1 | | 0.4 | | 0.04 | | | 0.3 | 0.2 | | 0.02 | | - | - | |  |
| Posterior cingulate cortex | 0.5 | | 0.1 | | 0.02 | | | -0.3 | 0.2 | | 0.01 | | - | - | |  |
| Precentral gyrus | 0.5 | | 0.1 | | 0.02 | | | 0.3 | 0.2 | | 0.02 | | - | - | |  |
| Precuneus | -0.04 | | 0.5 | | 0.05 | | | 0.1 | 0.3 | | 0.03 | | - | - | |  |
| Rostral anterior cingulate cortex | **0.8** | | **0.007** | | **0.002** | | | 0.2 | 0.3 | | 0.02 | | **1.6** | **0.05** | |  |
| Rostral middle frontal cortex | **0.6** | | **0.05** | | **0.01** | | | 0.02 | 0.5 | | 0.05 | | 1.2 | 0.1 | |  |
| Superior frontal gyrus | **0.8** | | **0.01** | | **0.003** | | | 0.1 | 0.4 | | 0.04 | | **1.7** | **0.04** | |  |
| Superior parietal gyrus | 0.3 | | 0.2 | | 0.03 | | | 0.3 | 0.2 | | 0.01 | | - | - | |  |
| Superior temporal gyrus | 0.6 | | 0.2 | | 0.01 | | | 0.1 | 0.4 | | 0.04 | | - | - | |  |
| Supramarginal gyrus | 0.4 | | 0.2 | | 0.02 | | | 0.4 | 0.1 | | 0.004 | | - | - | |  |
| Temporal pole | **0.7** | | **0.03** | | **0.01** | | | 0.2 | 0.3 | | 0.03 | | 1.1 | 0.1 | |  |
| Transverse temporal gyrus | **0.7** | | **0.03** | | **0.01** | | | 0.3 | 0.2 | | 0.01 | | 0.9 | 0.2 | |  |

BDNF – Brain Derived Neurotrophic Factor, 1-tailed p-values are reported, FDR p-value is derived using the Benjamini-Hochberg procedure, Fisher’s z-test for interaction a-lysis was performed only for significant correlations. Regions significantly correlating with sBDNF at p=0.05 are marked with yellow color.

**Supplementary Table 6. Subgroup analysis: Correlation between serum BDNF and normalized gray matter volume in subjects with and without history of depression**

| Region of Interest | Subjects without history ofdepression (n=8) | | | Subjects with history ofdepression (n=12) | | | Interaction analysis | |
| --- | --- | --- | --- | --- | --- | --- | --- | --- |
|  | Pearson’s correlation | p-value | p_FDR_ 0.05 | Pearson’s correlation | p-value | p_FDR_  0.05 | Fisher’s z | p-value |
| Left hemisphere | | | | | | | | |
| Bank of the superior temporal sulcus | -0.5 | 0.09 | 0.01 | 0.2 | 0.3 | 0.04 | - | - |
| Caudal anterior cingulate | 0.2 | 0.3 | 0.03 | **0.5** | **0.04** | **0.01** | -0.6 | 0.3 |
| Caudal middle frontal | -0.02 | 0.5 | 0.05 | 0.1 | 0.4 | 0.04 | - | - |
| Cuneus | -0.03 | 0.5 | 0.05 | 0.4 | 0.1 | 0.02 | - | - |
| Entorhinal cortex | 0.2 | 0.3 | 0.03 | 0.5 | 0.1 | 0.01 | - | - |
| Frontal pole | 0.2 | 0.4 | 0.04 | 0.3 | 0.2 | 0.03 | - | - |
| Fusiform gyrus | 0.2 | 0.4 | 0.04 | **0.5** | **0.04** | **0.01** | -0.9 | 0.2 |
| Inferior parietal gyrus | -0.6 | 0.08 | 0.01 | 0.4 | 0.1 | 0.02 | - | - |
| Inferior temporal gyrus | -0.4 | 0.2 | 0.02 | 0.2 | 0.2 | 0.03 | - | - |
| Insula | **0.8** | **0.01** | **0.002** | 0.1 | 0.3 | 0.04 | 1.6 | 0.1 |
| Isthmus cingulate cortex | -0.2 | 0.3 | 0.03 | 0.3 | 0.2 | 0.02 | - | - |
| Lateral occipital sulcus | -0.4 | 0.2 | 0.02 | -0.1 | 0.4 | 0.04 | - | - |
| Lateral orbitofrontal cortex | 0.4 | 0.2 | 0.02 | 0.2 | 0.3 | 0.03 | - | - |
| Lingual gyrus | 0.1 | 0.4 | 0.04 | 0.1 | 0.4 | 0.04 | - | - |
| Medial orbitofrontal cortex | **0.7** | **0.04** | **0.01** | **0.7** | **0.01** | **0.001** | -0.01 | 0.5 |
| Middle temporal gyrus | **-0.8** | **0.008** | **0.001** | 0.3 | 0.2 | 0.02 | **-2.6** | **0.005** |
| Paracentral gyrus | -0.2 | 0.3 | 0.03 | -0.3 | 0.1 | 0.02 | - | - |
| Parahippocampal gyrus | 0.2 | 0.4 | 0.04 | 0.1 | 0.4 | 0.05 | - | - |
| Pars opercularis | -0.01 | 0.5 | 0.05 | **0.5** | **0.05** | **0.01** | - | - |
| Pars orbitalis | -0.03 | 0.5 | 0.05 | 0.1 | 0.4 | 0.05 | - | - |
| Pars triangularis | 0.2 | 0.4 | 0.04 | 0.1 | 0.4 | 0.04 | - | - |
| Pericalcarine cortex | **0.6** | **0.05** | **0.01** | 0.4 | 0.1 | 0.01 | 0.5 | 0.3 |
| Postcentral gyrus | **0.6** | **0.05** | **0.01** | 0.4 | 0.1 | 0.02 | 0.5 | 0.3 |
| Posterior cingulate cortex | 0.5 | 0.1 | 0.01 | **0.6** | **0.01** | **0.004** | 0.3 | 0.4 |
| Precentral gyrus | -0.02 | 0.5 | 0.05 | -0.01 | 0.5 | 0.05 | - | - |
| Precuneus | 0.6 | 0.1 | 0.01 | **0.7** | **0.01** | **0.003** | -0.1 | 0.5 |
| Rostral anterior cingulate cortex | 0.4 | 0.2 | 0.02 | **0.6** | **0.02** | **0.01** | -0.6 | 0.3 |
| Rostral middle frontal cortex | 0.3 | 0.2 | 0.02 | 0.2 | 0.2 | 0.03 | - | - |
| Superior frontal gyrus | -0.6 | 0.1 | 0.01 | 0.1 | 0.4 | 0.05 | - | - |
| Superior parietal gyrus | -0.5 | 0.1 | 0.02 | 0.3 | 0.2 | 0.02 | - | - |
| Superior temporal gyrus | 0.3 | 0.2 | 0.03 | 0.2 | 0.3 | 0.04 | - | - |
| Supramarginal gyrus | 0.1 | 0.4 | 0.04 | 0.3 | 0.2 | 0.02 | - | - |
| Temporal pole | 0.4 | 0.2 | 0.02 | -0.02 | 0.5 | 0.05 | - | - |
| Transverse temporal gyrus | 0.3 | 0.3 | 0.03 | 0.2 | 0.3 | 0.03 | - | - |
| Right hemisphere | | | | | | | | |
| Banks of the superior temporal sulcus | -0.3 | 0.2 | 0.03 | -0.1 | 0.4 | 0.04 | - | - |
| Caudal anterior cingulate | 0.1 | 0.4 | 0.04 | -0.2 | 0.3 | 0.03 | - | - |
| Caudal middle frontal | 0.5 | 0.1 | 0.01 | 0.1 | 0.4 | 0.05 | - | - |
| Cuneus | 0.2 | 0.4 | 0.04 | 0.1 | 0.4 | 0.04 | - | - |
| Entorhinal cortex | 0.2 | 0.4 | 0.04 | -0.2 | 0.2 | 0.03 | - | - |
| Frontal pole | 0.2 | 0.4 | 0.04 | 0.3 | 0.2 | 0.02 | - | - |
| Fusiform gyrus | -0.3 | 0.3 | 0.03 | 0.2 | 0.2 | 0.03 | - | - |
| Inferior parietal gyrus | 0.04 | 0.5 | 0.05 | 0.2 | 0.3 | 0.04 | - | - |
| Inferior temporal gyrus | **-0.7** | **0.04** | **0.01** | **0.7** | **0.005** | **0.001** | **-3.0** | **0.002** |
| Insula | **0.7** | **0.02** | **0.003** | 0.2 | 0.3 | 0.03 | 1.34 | 0.1 |
| Isthmus cingulate cortex | -0.1 | 0.4 | 0.04 | **0.6** | **0.01** | **0.004** | -1.6 | 0.1 |
| Lateral occipital sulcus | 0.2 | 0.3 | 0.03 | 0.4 | 0.1 | 0.01 | - | - |
| Lateral orbitofrontal cortex | 0.3 | 0.2 | 0.03 | 0.4 | 0.1 | 0.02 | - | - |
| Lingual gyrus | 0.2 | 0.4 | 0.04 | 0.3 | 0.2 | 0.02 | - | - |
| Medial orbitofrontal cortex | 0.5 | 0.1 | 0.02 | **0.5** | **0.03** | **0.01** | -0.2 | 0.4 |
| Middle temporal gyrus | -0.5 | 0.1 | 0.02 | 0.4 | 0.1 | 0.01 | - | - |
| Paracentral gyrus | -0.03 | 0.5 | 0.05 | **-0.5** | **0.03** | **0.01** | 1.0 | 0.2 |
| Parahippocampal gyrus | -0.3 | 0.2 | 0.03 | -0.1 | 0.4 | 0.04 | - | - |
| Pars opercularis | -0.1 | 0.4 | 0.04 | 0.5 | 0.1 | 0.01 | - | - |
| Pars orbitalis | 0.4 | 0.2 | 0.02 | 0.3 | 0.2 | 0.02 | - | - |
| Pars triangularis | -0.4 | 0.2 | 0.02 | **0.6** | **0.02** | **0.01** | **-2.0** | **0.02** |
| Pericalcarine cortex | **0.7** | **0.03** | **0.00** | 0.3 | 0.2 | 0.03 | 1.0 | 0.2 |
| Postcentral gyrus | 0.2 | 0.3 | 0.03 | -0.1 | 0.4 | 0.04 | - | - |
| Posterior cingulate cortex | -0.3 | 0.2 | 0.02 | **0.7** | **0.01** | **0.002** | **-2.0** | **0.02** |
| Precentral gyrus | 0.5 | 0.1 | 0.01 | 0.5 | 0.1 | 0.01 | - | - |
| Precuneus | 0.3 | 0.2 | 0.03 | 0.3 | 0.1 | 0.02 | - | - |
| Rostral anterior cingulate cortex | -0.2 | 0.4 | 0.04 | -0.2 | 0.3 | 0.04 | - | - |
| Rostral middle frontal cortex | 0.3 | 0.2 | 0.02 | 0.1 | 0.4 | 0.04 | - | - |
| Superior frontal gyrus | 0.3 | 0.3 | 0.03 | 0.04 | 0.5 | 0.05 | - | - |
| Superior parietal gyrus | **0.6** | **0.05** | **0.01** | 0.3 | 0.2 | 0.03 | 0.9 | 0.2 |
| Superior temporal gyrus | **0.7** | **0.04** | **0.01** | 0.2 | 0.3 | 0.03 | 1.1 | 0.1 |
| Supramarginal gyrus | **0.7** | **0.03** | **0.004** | 0.5 | 0.1 | 0.01 | 1.1 | 0.1 |
| Temporal pole | **0.8** | **0.01** | **0.001** | 0.3 | 0.2 | 0.03 | 1.3 | 0.1 |
| Transverse temporal gyrus | 0.5 | 0.1 | 0.01 | 0.2 | 0.3 | 0.03 | - | - |
|  |  |  |  |  |  |  |  |  |

BDNF – Brain Derived Neurotrophic Factor, 1-tailed p-values are reported, FDR p-value is derived using the Benjamini-Hochberg procedure, Fisher’s z-test for interaction a-lysis was performed only for significant correlations. Regions significantly correlating with sBDNF at p=0.05 are marked with yellow color.
